# *In Vivo* Phenotyping of Tumor Metabolism in a Canine Cancer Patient with Simultaneous ^18^F-FDG-PET and Hyperpolarized ^13^C-Pyruvate Magnetic Resonance Spectroscopic Imaging (hyperPET): Mismatch Demonstrates that FDG may not Always Reflect the Warburg Effect

**DOI:** 10.3390/diagnostics5030287

**Published:** 2015-06-26

**Authors:** Henrik Gutte, Adam E. Hansen, Majbrit M.E. Larsen, Sofie Rahbek, Helle H. Johannesen, Jan Ardenkjaer-Larsen, Annemarie T. Kristensen, Liselotte Højgaard, Andreas Kjaer

**Affiliations:** 1 Department of Clinical Physiology, Nuclear Medicine & PET and Cluster for Molecular Imaging, Rigshospitalet, University of Copenhagen, Copenhagen 2100, Denmark; E-Mails: gutte@sund.ku.dk (H.G.); adam.espe.hansen@regionh.dk (A.E.H.); sofie89falko@gmail.com (S.R.); helle.hjorth.johannesen.01@regionh.dk (H.H.J.); liselotte.hoejgaard@regionh.dk (L.H.); 2 Department of Veterinary Clinical and Animal Sciences, Faculty of Health and Medical Sciences, University of Copenhagen, Frederiksberg C 2000, Denmark; E-Mails: majbritt.larsen@sund.ku.dk (M.M.E.L.); atk@sund.ku.dk (A.T.K.); 3 Department of Electrical Engineering, Technical University of Denmark, Lyngby 2800, Denmark; E-Mail: JanHenrik.ArdenkjaerLarsen@ge.com; 4 GE Healthcare, Brøndby 2605, Denmark

**Keywords:** cancer, dynamic nuclear polarization, hyperpolarized, ^13^C-pyruvate, MR, ^18^F-FDG-PET, PET/MR, molecular imaging, hyperPET

## Abstract

In this communication the mismatch between simultaneous ^18^F-FDG-PET and a ^13^C-lactate imaging (hyperPET) in a biopsy verified squamous cell carcinoma in the right tonsil of a canine cancer patient is shown. The results demonstrate that ^18^F-FDG-PET may not always reflect the Warburg effect in all tumors.

**Figure 1:**
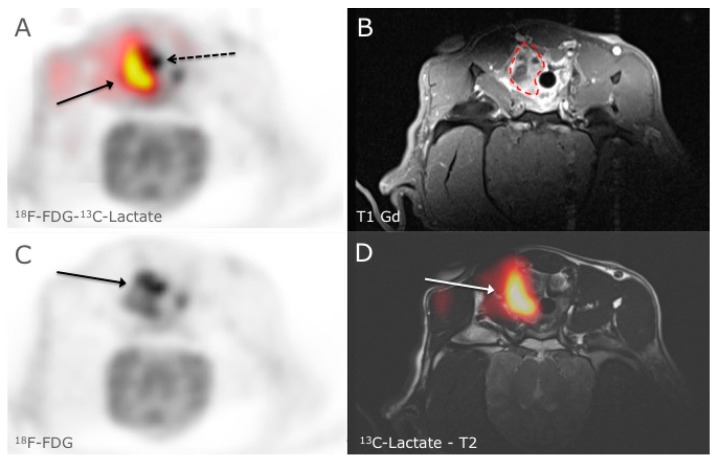
HyperPET is a new *in vivo* imaging modality that consists of combining a PET scan with magnetic resonance spectroscopic imaging (MRSI) of hyperpolarized ^13^C-pyruvate made possible by integrated hybrid PET/MRI systems [1]. The metabolism of cancer cells is characterized by a shift to glycolysis with production of lactate even in the presence of sufficient oxygen, this phenomenon is also known as the Warburg effect [2,3,4]. With the introduction of hyperpolarized ^13^C-pyruvate/^13^C-lactate MRSI it is probably now possible to directly study the metabolism of lactate and Warburg effect in real time. This is opposed to imaging with ^18^F-FDG PET scan alone, which demonstrates the Warburg effect only indirectly through increased glucose utilization and uptake. In Figure 1, ^18^F-FDG-PET and ^13^C-lactate MRSI in a spontaneous canine tumor is shown. A clear mismatch between ^18^F-FDG uptake and ^13^C-lactate production is seen. In an axial slice of the neck in a canine cancer patient with a biopsy verified squamous cell carcinoma in the right tonsil, we noticed in panel **A** clear discrepancy between the ^18^F-FDG-PET (^18^F-FDG activity is shown in grey scale and the dashed arrow points at the margin of tumor) and the ^13^C-lactate production (red to yellow color corresponds to the ^13^C-Lactate production and the arrow points to the margin of tumor) in a large heterogeneous tumor. ^18^F-FDG uptake in the tumor was variable in the tumor (panel **C**) and corresponded to the anatomical MR images in that high ^18^F-FDG levels paralleled the uptake of Gadolinium in the T1 sequence (panel **B**, dashed line outlines the contour of the tumor). However ^13^C-lactate did not correspond to the ^18^F-FDG uptake, especially in the more profound region of the tumor where we demonstrated a large production of ^13^C-lactate indicating higher degree of glycolysis (panel **D**). The Ethics and Administrative Committee, Department of Veterinary Clinical and Animal Sciences, Faculty of Health and Medical Sciences, University of Copenhagen approved the study. Whereas ^18^F-FDG-PET has generally been accepted as an indicator of the Warburg effect the hyperPET imaging of a canine tumor demonstrates that this may not always be the case. Accordingly, the new technique of hyperPET that we recently introduced can expose such diversity in metabolism. We suggest that hyperPET may become a valuable tool for better phenotyping of tumors to be used for prognostication, treatment planning and response monitoring.
